# Supplementary material for: Offshore freshened groundwater in the Pearl River estuary and shelf as a significant water resource
Source: Nat Commun. 2023 Jun 24;14:3781. doi: 10.1038/s41467-023-39507-0 (PMC10290674; doi:10.1038/s41467-023-39507-0)
Supplement: Supplementary file 1 — Supplementary Information [file 41467_2023_39507_MOESM1_ESM.pdf]

— Supplementary material for —

Offshore freshened groundwater in the Pearl River estuary and shelf as a significant water resource

Chong Sheng [1], Jiu Jimmy Jiao [1, 2, 3 *], Xin Luo [1, 2, 3], Jinchao Zuo [2], Lei Jia [3], Jinghe Cao [4]

[1] Department of Earth Sciences, The University of Hong Kong, Hong Kong, China

[2] The University of Hong Kong, Shenzhen Institution of Research and Innovation (SIRI), Shenzhen, China

[3] Southern Marine Science and Engineering Guangdong Laboratory (Zhuhai), Zhuhai, China

[4] Guangzhou Marine Geological Survey, China Geological Survey, Guangzhou, China

[5] Key Laboratory of Ocean and Marginal Sea Geology, South China Sea Institute of Oceanology, Innovation Academy of South China Sea Ecology and Environmental Engineering, Chinese Academy of Sciences, Guangzhou, China

Corresponding author: Jiu Jimmy Jiao, jjiao@hku.hk

This supplementary PDF file includes:

Sections Text S1 to S5

Figures S1 to S7

Tables S1 to S3

References

Text S1. Schematic diagram of a Rhizon sampler extracting porewater from offshore holes and measured salinity profiles.

Generally, a Rhizon sampler consists of four parts as mentioned in methods section of the manuscript. The thin, porous tube is inserted directly into an intact sediment core, and a 20 mL syringe is attached to the connector. The vacuum in the syringe is the main driving force for extraction of the porewater from the sediment core. Porewater then passes from the sediment through the porous tube and flexible hose into the collection syringe (20 mL). Furthermore, a three-way valve is added between the connector and syringe to facilitate multiple samplings in one position (Figure S1a). Given a sufficiently small tube pore size (0.15 μm), the Rhizon sampler also serves as a filter, removing microbial and colloidal particles. Before the anterior tube (with a hydrophilic membrane) is inserted into the core, a portable drill is used to make a small hole (5 mm) in the core barrel.

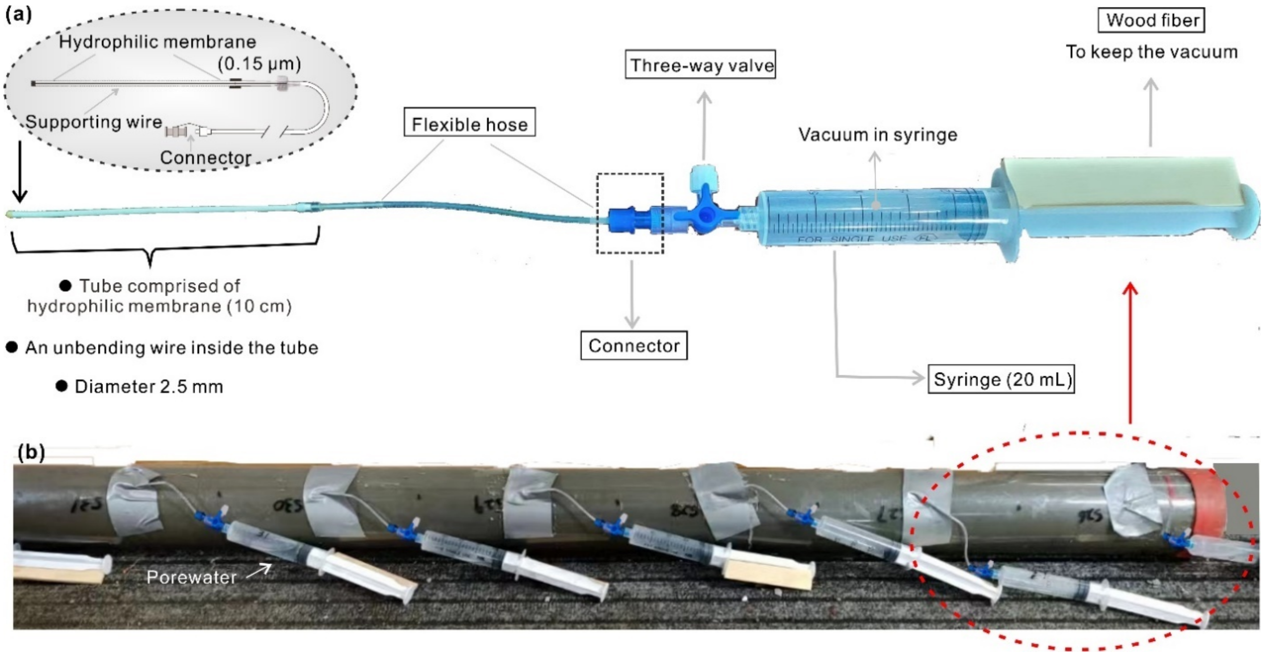

Figure S1. Schematic diagram of a Rhizon sampler (a) and the devices used for the porewater extraction from an offshore core (b) in the laboratory of the ocean drilling ship *Haiyang Dizhi*-10, The adhesive tape is used to prevent leakage of porewater and dissolved gas.

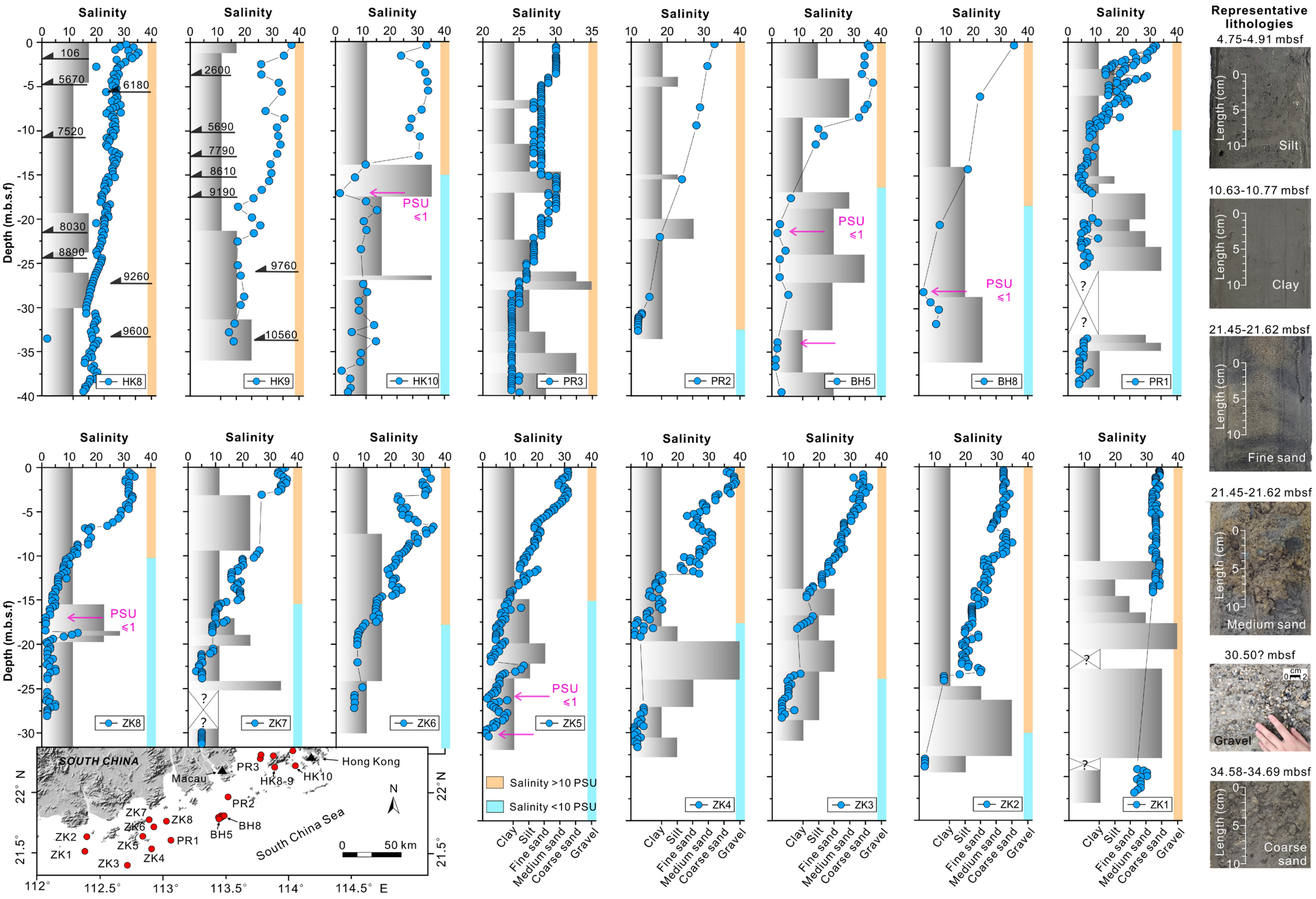

Figure S2. Geology and salinity profiles of offshore boreholes in the PRE and adjacent continental shelf. Selected images of representative lithologies in borehole PR3. Data were missing in some sections of other boreholes because of low recovery rates in sections with coarse materials

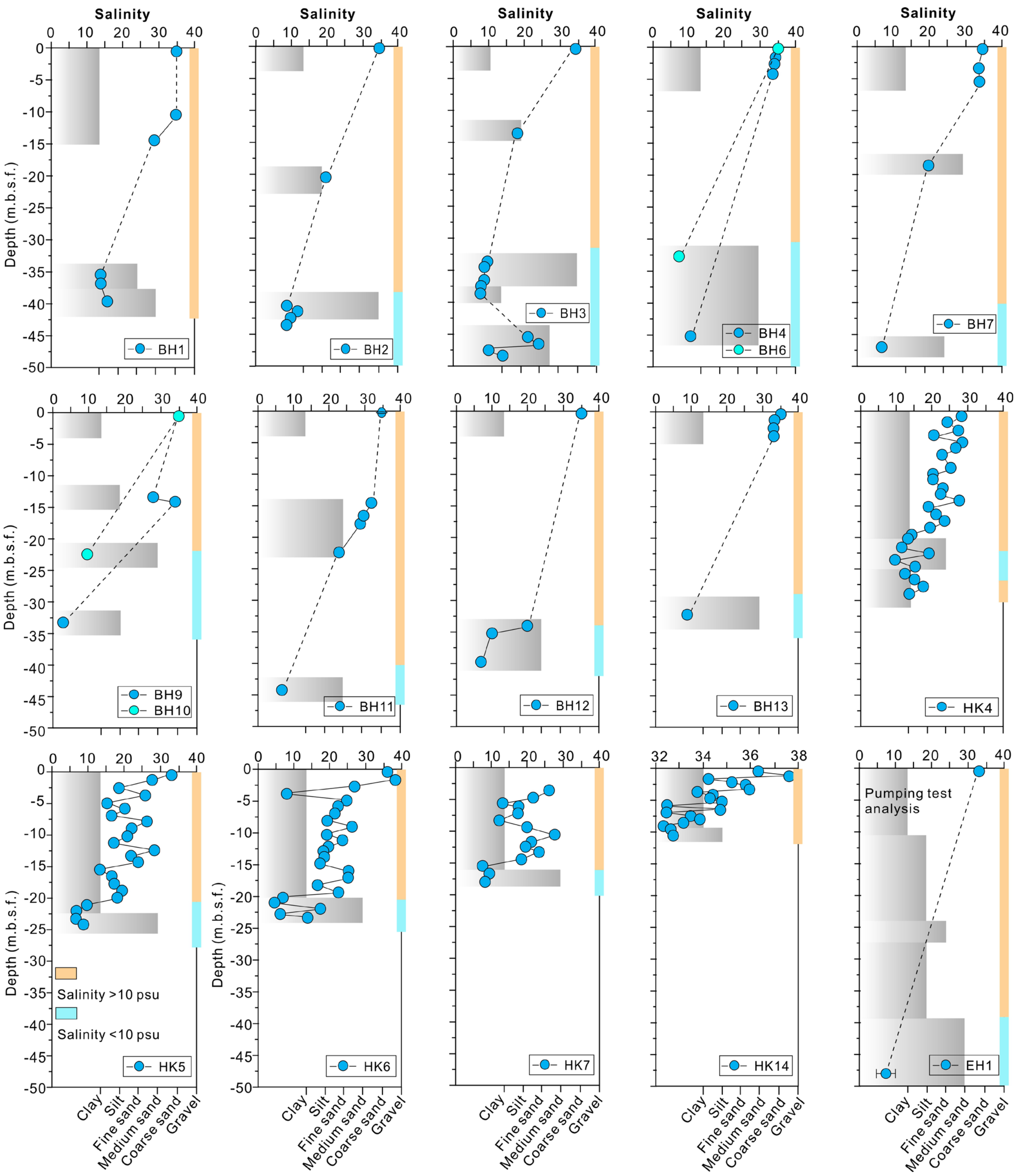

shelf and carried out a pumping test (site EH1) on the East Artificial Island of the Hong Kong-Zhuhai-Macao Bridge (in the Pearl River Estuary). The limited porewater samples from offshore boreholes BH1-13 in the study area were provided by the program of Sea Sand Exploration in the Northern South China Sea from not specifically for our research for porewater.

Table S1. *Comparison of typical indices of water quality of relatively fresh OFG with surrounding seawater in PRE and adjacent shelf.*

Samples	Depth (mbsf)	pH	Salinity (psu)	Major ions and nutrients (mg L^{-1})								Heavy metals and trace elements (ug L^{-1})							
				Na^+	K^+	Mg^{2+}	Ca^{2+}	Cl^-	SO_4^{2-}	NH_4^+	NO_x^-	B	Ba	Cu	Fe	Mn	Ni	Sr*	Zn
Standards of DW*	-	***6.5-8.5***	***1.0***	***200***	***20***	***50***	***100***	***250***	***250***	***0.5***	***20****	***500***	***700***	***1000***	***300***	***100***	***20***	***4000***	**1000**
SWr-PR1	-	***7.6***	***33.0***	***10628.2***	***375.7***	***963.4***	***161.6***	***19117.3***	***3155.1***	***3.5***	***9.8***	***2059.1***	***n.a.***	***n.a.***	***104.4***	***n.a.***	***n.a.***	***3749.8***	**n.a.**
PR1-S1	15.4	7.9	4.0	1582.0	114.4	114.3	44.3	2393.9	279.5	29.3	14.0	916.2	37.8	n.a.	385.8	375.7	5.3	1045.3	197.9
PR1-S2	21.9	7.8	5.0	1579.0	111.8	135.1	151.5	2573.8	125.6	35.2	40.6	994.8	82.8	5.3	640.0	236.0	5.2.	1143.7	190.7
PR1-S3	25.6	7.9	6.0	1496.8	87.3	136.6	118.8	2522.4	3.3	24.0	22.6	1017.5	136.4	n.a.	177.7	54.7	5.6	1513.4	141.8
PR1-S4	33.8	7.6	6.0	1496.8	69.0	136.6	118.8	2522.4	3.3	30.4	7.0	765.2	58.8	n.a.	1902.1	59.0	n.a.	765.1	42.2
PR1-S5	37.9	8.0	5.0	1586.6	63.9	169.4	193.0	2698.0	20.4	17.6	5.6	759.8	136.7	n.a.	1196.5	212.0	n.a.	1697.0	242.6
ZK8-S1	15.1	6.9	4.0	1546.7	66.1	170.1	107.6	3174.8	146.4	6.6	2.3	125.9	491.8	11.4	159.0	1800.2	5.5	1109.3	7.2
ZK8-S2	20.0	7.0	2.0	740.1	23.2	183.3	356.9	2084.2	158.1	4.9	1.7	85.8	707.7	6.3	13.9	2233.0	3.4	1652.1	9.8
ZK8-S3	25.0	7.8	1.0	307.9	14.5	117.4	245.4	980.7	179.8	4.2	2.4	57.7	56.1	n.a.	n.a.	893.7	1.6	1117.5	8.8
ZK8-S4	30.1	7.9	1.0	219.7	13.9	119.7	263.1	902.7	276.2	4.6	2.1	66.3	n.a.	n.a.	n.a.	875.7	0.9	1298.3	31.7
ZK5-S1	15.1	7.4	9.0	3096.0	117.5	340.9	331.3	6382.5	756.0	34.5	11.5	261.0	77.3	55.8	n.a.	2171.5	4.6	4025.2	16.6
ZK5-S2	20.1	7.2	7.0	2352.3	89.6	229.1	299.1	4499.7	401.3	32.4	16.2	184.2	122.6	55.3	n.a.	1224.2	3.1	4529.7	n.a.
ZK5-S3	25.3	7.7	4.0	1326.9	75.5	151.9	397.9	2917.2	705.0	38.0	8.4	171.3	119.3	58.5	n.a.	1083.4	2.9	7970.8	24.0
ZK5-S4	30.0	7.4	1.0	762.2	52.6	75.7	281.6	1911.7	300.7	31.7	7.6	156.7	58.9	51.1	n.a.	912.8	1.7	6489.2	11.7
SW-PR2	-	***8.1***	***33.0***	***10557.5***	***375.2***	***1178.7***	***320.4***	***17236.5***	***2263.3***	***13.5***	***79.8***	***2455.1***	***n.a.***	***n.a.***	***40.0***	***n.a.***	***n.a***	***4527.1***	**n.a.**
PR2-S1	30.6	8.0	13.0	3523.5	129.2	288.3	50.7	6116.0	3.4	323.7	35.8	1803.3	123.5	n.a.	4107.5	106.1	24.9	2315.7	47.0
PR2-S2	31.1	8.1	12.0	3502.3	128.4	295.7	55.5	6179.1	5.4	297.8	36.4	1211.4	101.1	n.a.	4131.2	81.5	25.9	1518.6	148.9
PR2-S3	32.0	8.0	12.0	3284.9	120.3	283.6	62.1	5934.5	0.1	353.5	39.2	1258.1	143.1	n.a.	6527.8	88.6	40.9.	1711.0	51.7
PR2-S4	32.7	7.9	12.0	3526.5	156.3	270.5	98.3	5276.2	100.3	331.8	85.4	1214.5	106.4	n.a.	1613.2	85.9	20.3	1547.9	16.3
BH5-S1	20.5	7.6	3.0	1722.6	100.1	120.7	150.3	2758.3	203.3	0.4	6.4	250.5	194.8	79.2	n.a.	3242.2	14.4	997.1	0.7
BH5-S2	26.5	7.8	3.0	1400.8	82.5	93.8	180.8	2348.3	151.9	2.1	3.2	153.6	277.7	79.2	n.a.	1901.3	3.9	1003.1	4.9
BH5-S3	34.6	7.5	2.0	708.3	24.3	120.4	398.9	1497.0	542.4	4.2	5.4	60.7	5.1	63.3	n.a.	1378.7	1.7	1704.7	5.7
BH5-S4	36.6	7.4	1.0	400.1	15.7	81.5	283.5	952.8	333.4	3.8	2.8	29.4	65.6	57.6	n.a.	1068.7	2.1	1209.3	1.8
BH5-S5	44.7	7.6	1.0	100.7	11.2	20.9	103.0	345.9	76.4	2.9	2.4	40.4	n.a.	n.a.	21.1	140.9	1.7	577.4	0.6
BH5-S6	45.9	7.2	1.0	289.0	22.8	129.2	357.8	713.9	895.5	7.3	1.8	82.3	n.a.	n.a.	n.a.	681.4	85.7	1699.6	39.5
BH8-S1	28.5	7.6	1.0	419.0	17.1	8.1	173.2	941.7	146.8	3.6	1.6	65.3	1376.5	n.a.	n.a.	579.4	1.1	838.0	1.3
BH8-S2	29.5	7.5	4.0	1302.5	27.7	10.2	411.6	2740.9	376.4	7.9	6.8	114.5	1699.7	11.5	n.a.	544.1	25.5	1929.1	26.5
SW-PR3	-	***7.5***	***31.0***	***9518.7***	***339.9***	***1026.1***	***305.8***	***15471.4***	***2030.2***	***0.6***	***29.4***	***2299.5***	***n.a.***	***n.a.***	***60.8***	***n.a.***	***n.a.***	***4041.1***	**n.a.**

Note: SW, seawater. DW, drinking water, the standard mainly refers to Ministry of Health of China, but the limitation of heavy metal Sr refers to the U.S. EPA. The concentration of NO_x^- is counted by "N". n.a. means the concentration of the index is below 5.0 ppb or the detection threshold of the analytical method. Other heavy metals, i.e., Be, Cd, Cr, Co, Ga, Pb, Se, Te, Tl, and Bi with concentrations below this detection threshold are not listed.

Text S2. The OFG caused by decomposition of gas hydrates and dehydration of clay minerals in the South China Sea

In the northern continental shelf of the South China Sea, two other emplacement mechanisms can also contribute to a low-salinity anomalies in seabed porewater. Post - sedimentary alteration processes can lead to the release of fresh water and the formation of freshened water fronts in deeply buried marine sediments or in high pressure convergent margins (Figure S3a) [11]. The seabed unconsolidated sediments in the northern margin of the South China Sea are also rich in gas hydrates, and fresh water can be released by the decomposition of gas hydrates resulting in a low-salinity anomaly (Figure. S3b) [12]. However, the deviations in values of stable isotopic compositions of $\delta^{18}O$ and $\delta^{2}H$ caused by these two emplacement mechanisms are completely different from that of meteoric water as mentioned in the manuscript.

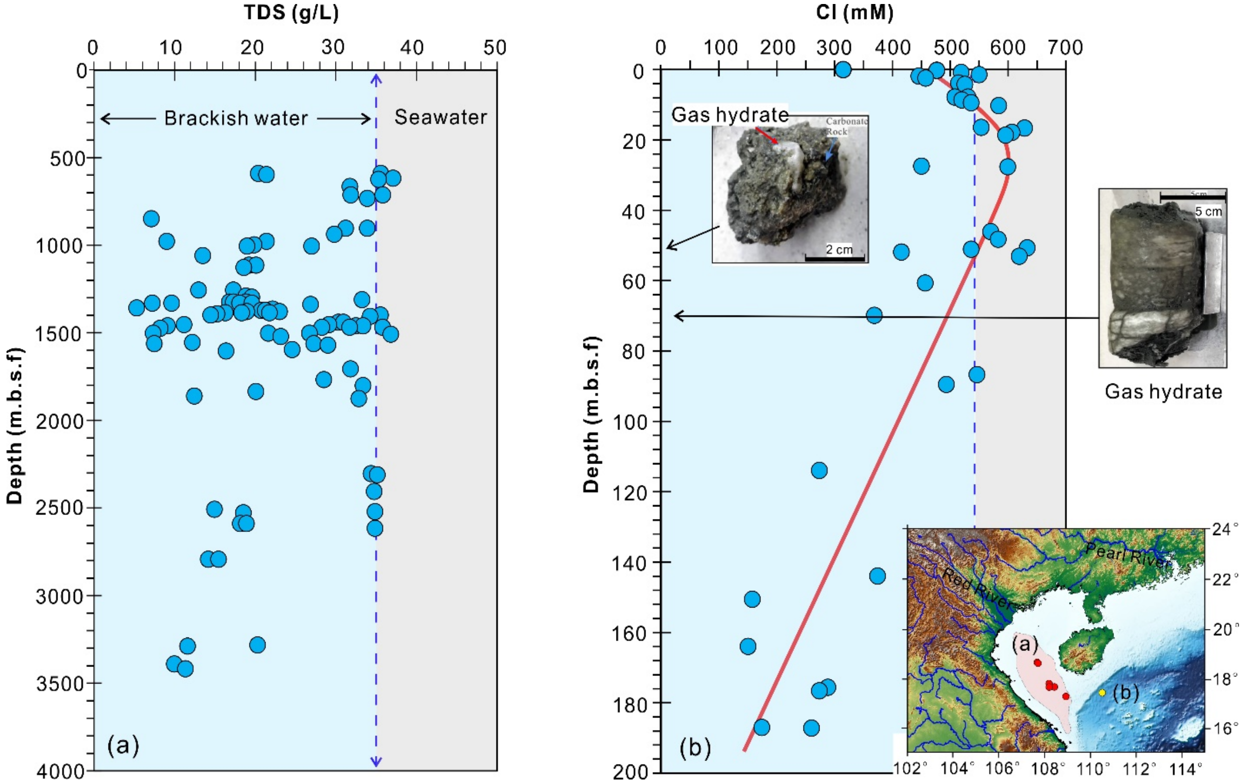

Figure S3. The OFG caused by the dehydration of deeply buried clay minerals and decomposition of buried gas hydrates in the northern South China Sea. (a) Yinggehai Basin; (b) Qiongdongnan basin.

Text S3. Reconstruction of paleochannels and Quaternary strata in the PRE and adjacent shelf.

Buried paleochannels show a typical characteristic of discontinuities and "V" or "U" downcutting in the sub-bottom seismic reflections. However, the Quaternary strata is delineated in the seismic reflections by continuous, high-amplitude and mid-strong reflections and labelled T20, which can be continuously tracked in the study area [1]. Some typical profiles can be found in previous studies [1, 2, 3, 4, 5].

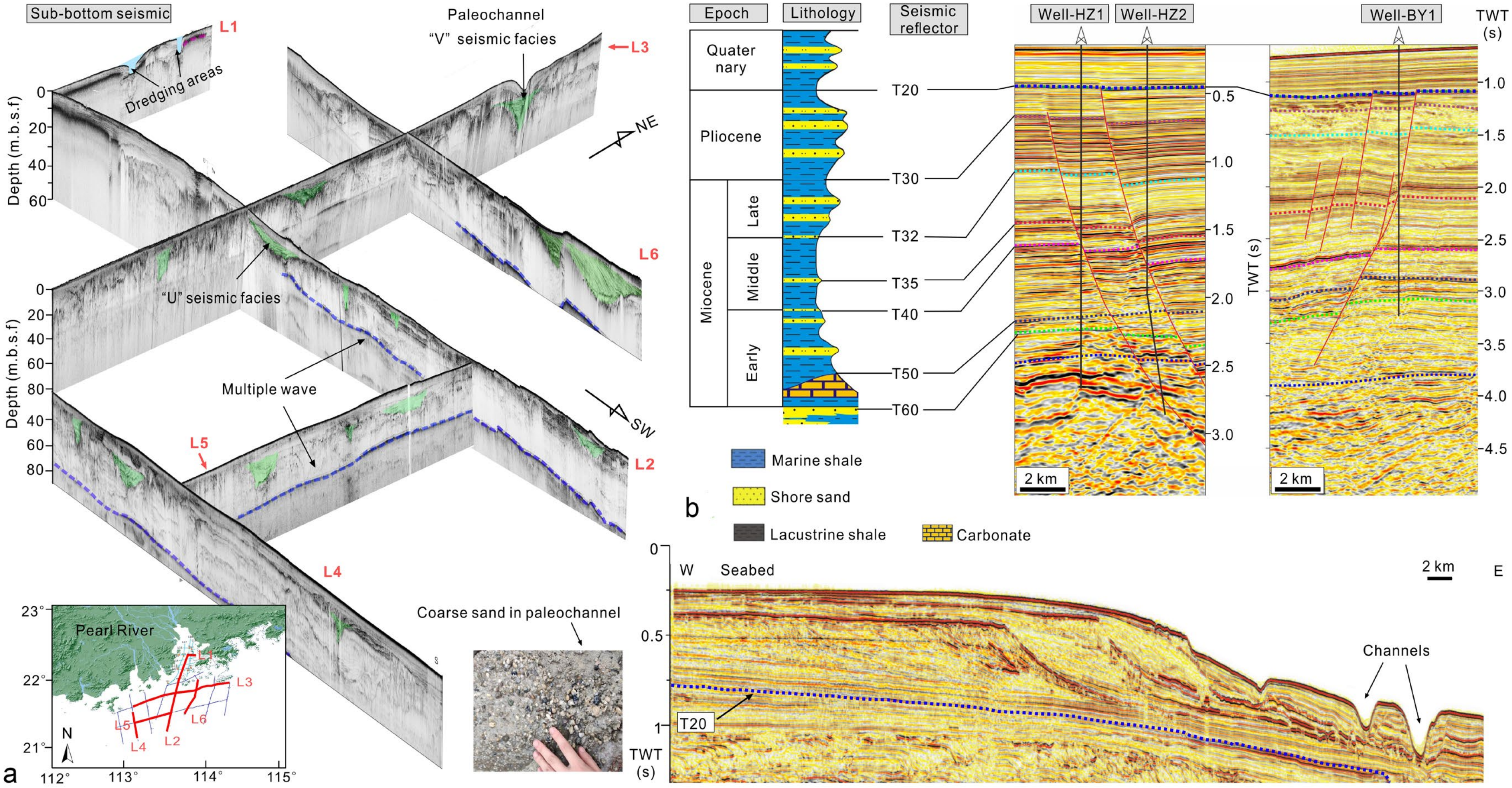

Figure S4. Interpreted results of high-resolution sub-bottom and multi-channel seismic profiles. (a) Typical paleochannel seismic facies classified in this study. (b) The comprehensive strata log diagram and reflection characteristics of Quaternary strata in seismic reflections [1].

Text S4. Reconstruction of sediments basement in the PRE and adjacent shelf.

The seismic-reflection survey to characterize the offshore faults and basement in the PRE and adjacent shelf was conducted by *research vessel "Haidiao*-6" in May 2015. A total of 13 two-dimensional, 24-channel seismic-reflection profiles were obtained, which included 10 lines in the northwest-to-southeast direction (S1-S8) with lengths between 115 and 145 km and three northeast-to-southwest trending profiles (W1-W3) with lengths exceeding 210 km (Figure S5). The seismic source was generated by an array of four Bolt 1500 L airguns with a total volume of 6000 in^3 operated at an air pressure of 2000 lbs in^{-2}. The airguns were shot at intervals of 80 s, which corresponds to approximately 200 m at the ship speed of 5 knots, towed at a depth of 10 m. The receiver array was a 75 m long 24-channel MicroEel Analog Seismic Solid Streamer produced by Geometrics with a group spacing of 3.125 m. The record length for each shot was 6 s, and a sampling rate of 1 ms was adopted. For navigation and shot timing, the GPS was used to record the position of each shot and the seismic data were recorded in SEG Y format [6].

The seismic reflection data were processed as follows: (1) geometry definition in trace header; (2) trace editing in seismic data sets; (3) automatic gain control with 0.5s window and amplitude compensation to get the true amplitude; (4) application of Butterworth bandpass filter of 10-15-35-40 Hz to enhance airgun signal and suppress noise, and (5) predictive deconvolution using the minimum lag of 0.06 s and maximum lag of 0.12 s to suppress the reverberation generated by the oscillating signal of the airgun. Finally, the depth of each measurement below seafloor was obtained based on the regional time-depth conversion empirical formula derived by the time-depth correction in the depth on the vertical seismic profile [6, 7]:

$$D_2 = 0.0002t^2 + 0.9549t - 154.01 \quad (1)$$

where D_2 is the depth below the seafloor (m), t is two-way travel time (ms), and R^2=0.9886 here.

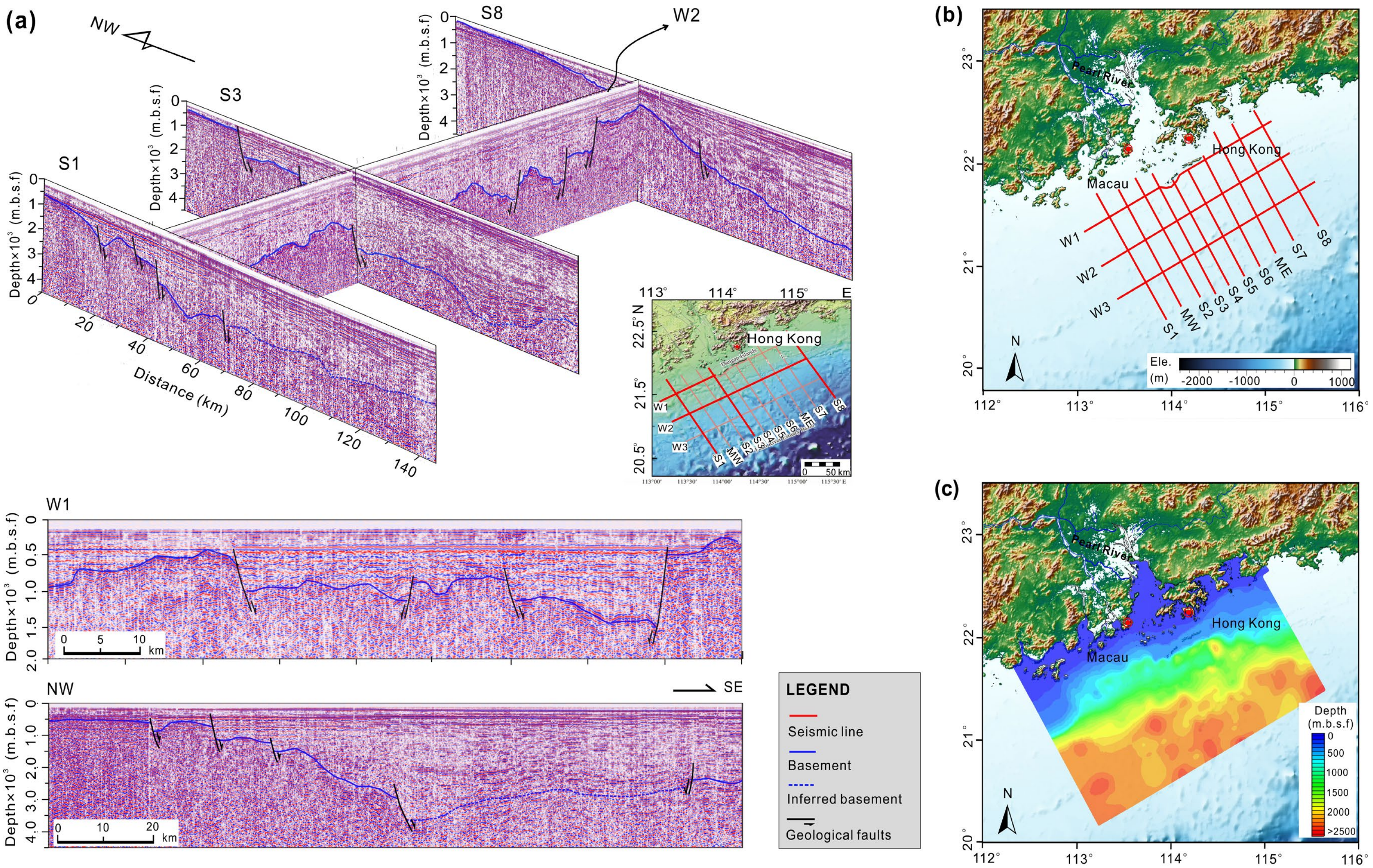

Figure S5. Interpreted results of seismic-reflection profiles in the PRE and adjacent continental shelf. (a) Typical profiles reflect the distribution of the sediment basement (blue lines) and faults (black lines). (b) Locations of the marine seismic lines collected in the PRE and adjacent continental shelf. (c) Spatial distribution map of the sediment basement depth as interpreted from the MCS profiles (red lines).

Text S5. Geostatistical model and paleo-hydrogeologic modelling settings.

In the offshore domain, the upper model cells and the rightmost column are assigned a specified head boundary, which equals the sea level elevation according to the reconstructed eustatic sea-level curve, with a concentration of 35 g L^{-1}. For nodes above sea level, we imposed a specified flux along the top boundary to simulate the groundwater recharge. As no long-term precipitation record exist for the Pearl River delta over the past 125 kyr, we chose to apply a constant uniform recharge of 1.23 mm d^{-1}, which is equal to about 25 % of the current long-term precipitation average close to similar previous study [8]. The variable-density groundwater flow and coupled salt transport modelling software SEAWAT is used to solve the simulations using the finite difference method.

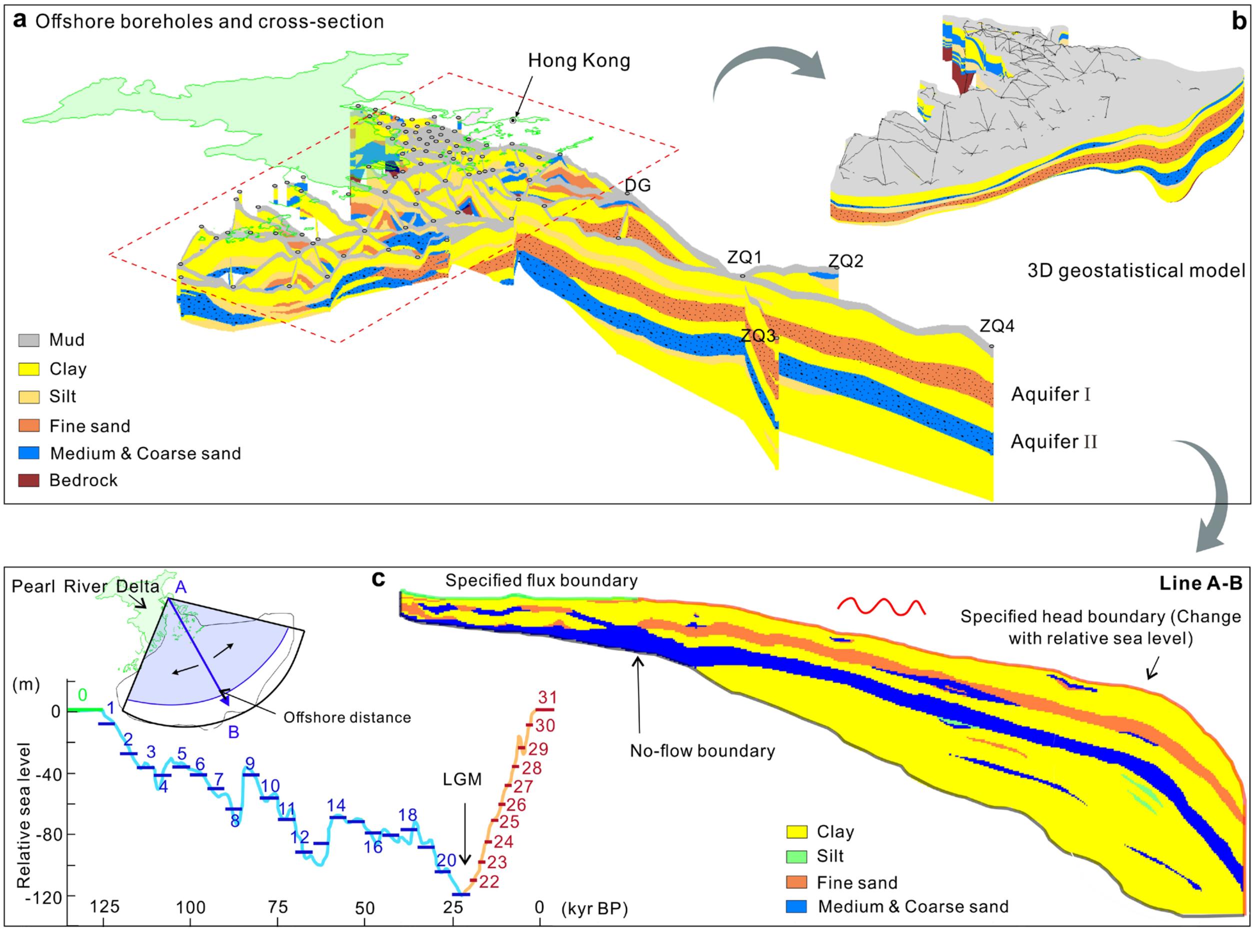

Figure S6. 3D geostatistical model for inner shelf and paleo-hydrogeological modelling settings. (a) Offshore geological boreholes, cross-sections, and the 3D geostatistical model for the inner shelf (~55 km offshore), (b) Boundary conditions and hydrogeological units used in the numerical model.

Table S2. *Main hydrogeologic parameters used for the base case of cross-section A-B in the study area.*

Hydrogeological units	Horizontal K_H (m s^{-1})	Anisotropy (K_H/K_v)	Porosity
Clay	1×10^{-9}	3	0.45
Silt	1×10^{-6}	3	0.4
Fine sand	5×10^{-5}	3	0.35
Medium & Coarse sand	2.5×10^{-4}	3	0.3
Longitudinal diversity	50 m		
Vertical diversity	0.5 m		
Molecular diffusion coefficient	8.64×10^{-5} m^2 d^{-1}		

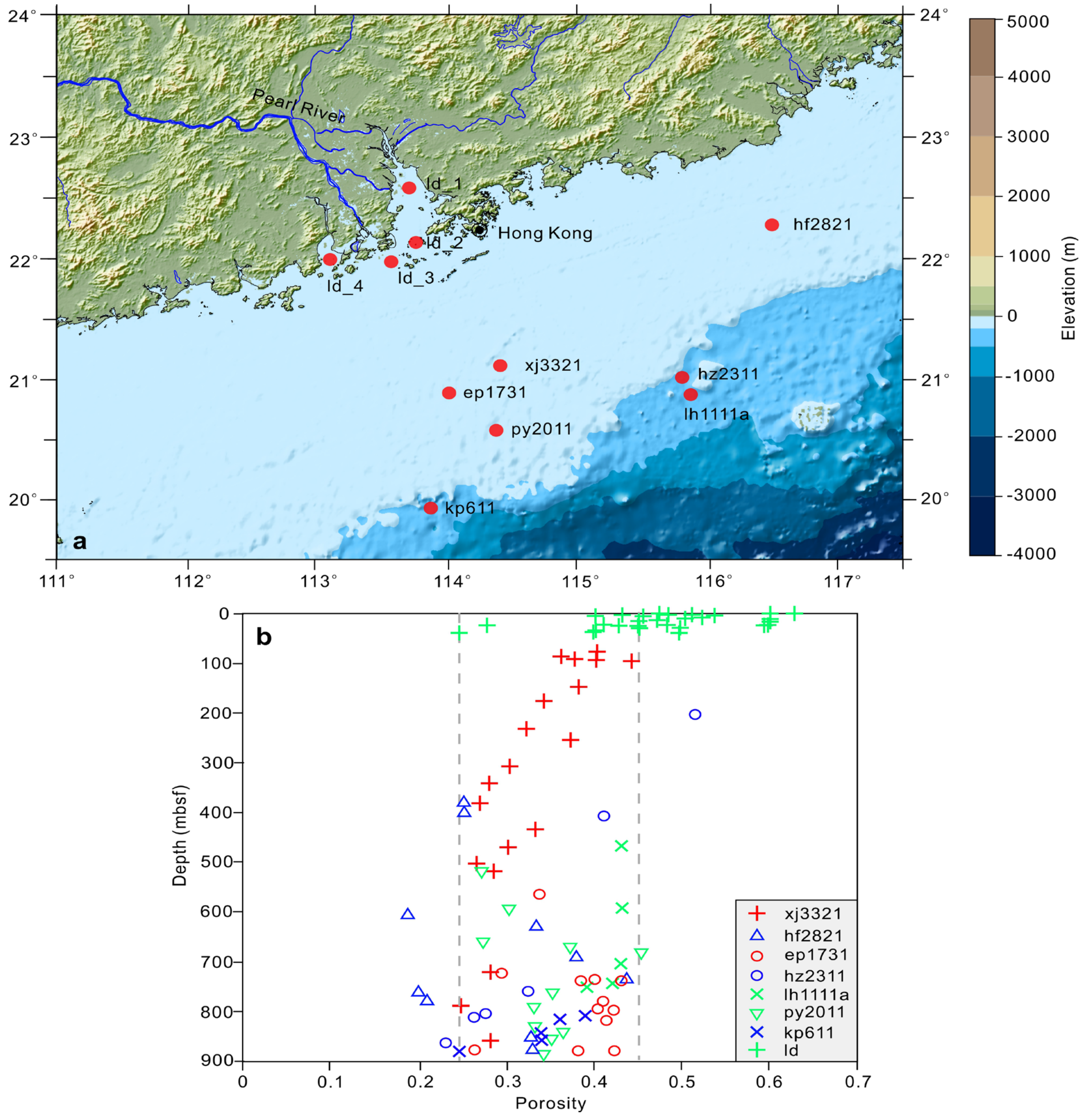

Figure S7. Porosity obtained from unconsolidated sediments in offshore boreholes from previous studies [2, 9, 10]. (a) The locations of the offshore boreholes in the PRE and adjacent continental shelf, (b) Porosity versus depth of the unconsolidated sediments obtained from these offshore boreholes.

Table S3. *Characteristics of OFG bodies recorded in estuarine regions and its adjacent continental shelves of the major rivers worldwide.*

Main rivers	Locations	River discharge (km^3 y^{-1})	Offshore distance (km)	Depth of top of OFG (m.b.s.f)	Mini. Salinity (g L^{-1})	Water depth (m)	Methods to identify OFG	Porosity (%)	Onshore connection	References
Yangtze River	East China Sea	950.0	50	~40	1.277	15	BH	n.a.	Unclear	Zhang, Zou [13]
Pearl River	South China Sea	343.0	55	0	<1.0	20~30	BH	30-45	Yes	This study
Red River	Northern Vietnam	135.0	>20	60	1.0	10~20	NM & OI	30-45	Yes	Larsen, Tran [14]
Mekong River	Southern Vietnam	504.0	~100	20~40	1.0	<100	NM & OI	25-50	Yes	Hung Van, Van Geer [15]
Rakaia River	New Zealand	2.7	60	< 50	< 10.	<110	BH & CSEM	40-60	Yes	Micallef, Person [16]
Waimakariri River	New Zealand	2.4	n.a.	n.a.	n.a.	<250	OI	n.a.	Unclear	Morgan and Mountjoy [17]
Rakaia River	New Zealand	6.4	n.a.	n.a.	n.a.	<250	OI	n.a.	Unclear	Morgan and Mountjoy [17]
Niger River	Nigeria	176.0	40	100	0.2	<100	BH	20-36	Yes	Oteri [18]
Llobregat River	Spain	0.5	5	~60	1.0	100	BH & OI	n.a.	Yes	Custodio [19]
Mississippi River	Louisiana (USA)	581.0	120	n.a.	<10.0	<30	BH	24-30	Yes	Osborn, Smith [20]
Ganges-Brahmaputra Rivers	Bangladesh & India	1202.0	~100	0	1.0	<200	NM	n.a.	Yes	Yu and Michael [21]
Suriname River	Suriname	151.0	90	0	1.0	50	BH & OI	n.a.	Yes	Groen, Velstra [22]
Tagliamento River	Northern Adriatic Sea	2.2	n.a.	n.a.	<10	< 25	BH	n.a.	yes	Giustiniani, Busetti [23]
Indian River	Delmarva Peninsula	n.a.	>1	5~10	5~7	10~20	BH	n.a.	yes	Krantz, Manheim [24]
Vouga River	Northwest Portugal	1.6	n.a.	n.a.	n.a.	< 60	BH & OI	n.a.	Yes	Condesso et al. [26]

Note. Methods to identify OFG include offshore boreholes (BH), numerical modelling (NM), onshore indicators (OI) mentioned by Post, Groen [25], or marine controlled-source electromagnetic methods (CSEM). n.a. means details not available.

References

1. Li G, Mei L, Ye Q, Pang X, Zheng J, Li W. Post-rift faulting controlled by different geodynamics in the Pearl River Mouth Basin, northern South China Sea margin. *Earth-Science Reviews* **237**, (2023).

2. He M*, et al.* Rapid post-rift tectonic subsidence events in the Pearl River Mouth Basin, northern South China Sea margin. *Journal of Asian Earth Sciences* **147**, 271-283 (2017).

3. Kong L, Chen H, Ping H, Zhai P, Liu Y, Zhu J. Formation pressure modeling in the Baiyun Sag, northern South China Sea: Implications for petroleum exploration in deep-water areas. *Marine and Petroleum Geology* **97**, 154-168 (2018).

4. Liu H*, et al.* Quaternary sequence stratigraphic evolution of the Pearl River Mouth Basin and controlling factors over depositional systems. *Marine Geology & Quaternary Geology* **39**, 25-37 (2019).

5. Qiu Y*, et al. Erosive process and sedimentary characteristics of the Quaternary sediments in the northern South China Sea*. Geological Press (2017).

6. Cao J, Xia S, Sun J, Zhao F, Wan K, Xu H. Offshore fault geometrics in the Pearl River Estuary, Southeastern China: evidence from seismic reflection data. *Journal of Ocean University of China* **17**, 799-810 (2018).

7. Li W, Wang P, Zhang C, Lu B. Researches on time-depth conversion of deep-seated basal strata of Pearl River Mouth basin. *Chinese Journal of Geophysics* **54**, 449-456 (2011).

8. Hung Van P, Van Geer FC, Bui Tran V, Dubelaar W, Oude Essink GHP. Paleo-hydrogeological reconstruction of the fresh-saline groundwater distribution in the Vietnamese Mekong Delta since the late Pleistocene. *Journal of Hydrology: Regional Studies* **23**, 100594 (2019).

9. Liang X, Jin M, Wang X, Zhang R. Evolution of paleo-groundwater dynamic field and its influence on oil-gas accumulation and dispersion.). China University of Geoscience (Wuhan) (1999).

10. Xia Z, Lin J, Zheng Z, Shi Y. *A comprehensive study of the marine geology and environment in the Pearl River Estuary*. Science Press (2015).

11. Xie X, Jiu JJ, Li S, Cheng J. Salinity variation of formation water and diagenesis reaction in abnormal pressure environments. *Science in China Series D: Earth Sciences* **46**, 269-284 (2003).

12. Ye J, Wei J, Liang J, Lu J, Lu H, Zhang W. Complex gas hydrate system in a gas chimney, South China Sea. *Marine and Petroleum Geology* **104**, 29-39 (2019).

13. Zhang Z, Zou L, Cui R, Wang L. Study of storage conditions of submarine freshwater resoureces and the submarine freshwater resources at north Zhoushan sea area. *Marine Science Bulletin* **30**, 47-52 (2011).

14. Larsen F, Tran LV, Van Hoang H, Tran LT, Christiansen AV, Pham NQ. Groundwater salinity influenced by Holocene seawater trapped in incised valleys in the Red River delta plain. *Nature Geoscience* **10**, 376-+ (2017).

15. Hung Van P, Van Geer FC, Bui Tran V, Dubelaar W, Oude Essink GHP. Paleo-hydrogeological reconstruction of the fresh-saline groundwater distribution in the Vietnamese Mekong Delta since the late Pleistocene. *Journal of Hydrology: Regional Studies* **23**, (2019).

16. Micallef A, *et al.* 3D characterisation and quantification of an offshore freshened groundwater system in the Canterbury Bight. *Nature Communications* **11**, 1372 (2020).

17. Morgan LK, Mountjoy JJ. Likelihood of offshore freshened groundwater in New Zealand.

Hydrogeology Journal, (2022).

18. Oteri AU. Electric log interpretation for the evaluation of salt water intrusion in the eastern Niger Delta. *Hydrological Sciences Journal* **33**, 19-30 (1988).

19. Custodio E. *Low Llobregat Aquifers: Intensive Development, Salinization, Contamination, and Management*.). Springer Berlin Heidelberg (2012).

20. Osborn NI, Smith SJ, Seger CH. Hydrogeology, distribution, and volume of saline groundwater in the southern midcontinent and adjacent areas of the United States. In: *Scientific Investigations Report*). United Stated Geological Survey (2013).

21. Yu X, Michael HA. Offshore pumping impacts onshore groundwater resources and land subsidence. *Geophysical Research Letters* **46**, 2553-2562 (2019).

22. Groen J, Velstra J, Meesters AGCS. Salinization processes in paleowaters in coastal sediments of Suriname: evidence from $d^{37}Cl$ analysis and diffusion modelling. *Journal of Hydrology* **234**, 1-20 (2000).

23. Giustiniani M*, et al.* Geophysical and Geological Views of Potential Water Resources in the North-Eastern Adriatic Sea. *Geosciences* **12**, 139 (2022).

24. Krantz DE, Manheim FT, Bratton JF, Phelan DJ. Hydrogeologic Setting and Ground Water Flow Beneath a Section of Indian River Bay, Delaware. *Ground Water* **42**, 1035-1051 (2004).

25. Post VE, Groen J, Kooi H, Person M, Ge S, Edmunds WM. Offshore fresh groundwater reserves as a global phenomenon. *Nature* **504**, 71-78 (2013).

26. Condesso de Melo MT, Carreira Paquete PM, Marques Da Silva MA. Evolution of the Aveiro Cretaceous aquifer (NW Portugal) during the Late Pleistocene and present day: evidence from chemical and isotopic data. *Geological Society*, London, Special Publications **189**, 139-54 (2001).
